# 

**Published:** 1898-01

**Authors:** 


### PERSONALS.

Dr. R. B. Plageman officiated as veterinarian to the Metropolitan Kennel Club Show in Brooklyn in November.

Dr. H. S. Perley, of St. Albans, Vt., has removed to Ottawa, Canada, and formed a copartnership with Government Inspector, Veterinarian A. E. James.

Dr. T. G. Risum, of Montevideo, Minn., has been away from home on a three months' trip.

Dr. A. F. Schrieber, of Philadelphia, is directing veterinarian of a specially kept dairy where a prepared koumyss is made for the market.

Dr. W. L. Zuill, of Philadelphia, in conjunction with his practice in human medicine, will manage a drugstore in the northern central part of the city.

Dr. John A. Pearson, of Philadelphia, is pursuing a post-graduate course at the Veterinary Department of the University of Pennsylvania.

Dr. Leonard Pearson, of Philadelphia, was prominent among those attending the Convention of Grangers at Harrisburg, Pennsylvania, in November.

Dr. S. E. Weber, of Lancaster, Pa., was a visitor to Philadelphia in November, in consultation with several of our city's specialists in surgery.

Dr. Leonard Pearson was on the ill-list for several days in November.

Dr. William Dougherty, of Baltimore, Md., was confined to his bed for some days in the latter part of November.

The veterinary inspectors at the recent Pittsburg horse-show were Drs. J. C. McNeil, G. H. Dunn, and J. Stewart Lacock.

Dr. A. W. Clement, of Baltimore, was among those who attended the Fasig sale of trotters in New York, and purchased the trotting-horse "Walter S.," 2.12½ mark.

Knock's cork-stone for stable-floors is the latest production. Said to be waterproof, fireproof, and noiseless, and a very slow wearer. It is composed of ground cork, ground tan-bark, ground peanut-shells, waste-paper and alum-water. This combination is baked in kilns.

Drs. J. W. Sallade, of Pottsville, and J. C. McNeil, of Pittsburg, were visitors to Philadelphia in December, attending the mid-winter examinations of the Pennsylvania State Board of Veterinary Medical Examiners.

Veterinarian James T. McAnulty, of Philadelphia, Vice-President of the National Horseshoers' Protective Association, is a frequent contributor to the columns of the *Horseshoers' Journal.*

Dr. A. Liautard holds the position of consulting veterinarian, and Dr. S. K. Johnson that of veterinarian to the Department of Health of New York City.

Dr. J. F. Jones, of Marietta, Ohio, was a visitor to the East during December, stopping at the JOURNAL office on his way homeward.

Dr. James Walsh, of New York City, has been added to the Scientific Committee of the National Master-shoers' Protective Association.

Dr. D. E. Salmon has an article in *The Feather* for November on "Mycosis of the Air-passages; Aspergillosis of Fowls."

Dr. W. G. Houck has recorded some interesting observations on rabies in his report printed in the yearly proceedings of the Board of Agriculture of Pennsylvania.

Dr. James Robertson, of Chicago, after four years of earnest, active work in the interest of the shoeing craft, has retired from the Scientific Committee.

Dr. D. E. Salmon is one of the Board of Directors of the National Poultry and Pigeon Association.

Dr. C. Stewart has been transferred in charge of work from Toledo, Iowa, under the Bureau of Animal Industry, to Kansas City, Mo.

Dr. A. T. Peters, of Lincoln, Neb., addressed the Nebraska Jersey Cattle-breeders' Association at Crete, Neb., in December.

Prof. A. Liautard, after a sojourn of two months in America, returned to join his family in sunny France. While here a neatly-bound copy of the college announcement for 1897–98 was sent to a number of the Alumni of the American Veterinary College.

Dr. Leonard Pearson was a visitor to the Capital, spending a portion of the holidays in the Congressional city.

Dr. E. P. Schaffter has been promoted and transferred to Toledo, Iowa, as chief inspector for that district.

Dr. J. C. Dustan, of Morristown, N. J., passed the 60th milestone of life in December, in the full vigor of health and with the zeal of a much younger man in the work of the profession coursing through his veins.

Veterinarian Owen Reist, of Kossuth, Ontario, holds the position of Sanitary Inspector for Waterloo township. This work includes a systematic inspection of all dairies and herds supplying milk to the towns of this district.

Veterinarian Hugh F. Doris, of Pittsburg, Pa., found himself in the hands of the law during December. The charge against him was exposing for sale watered milk, he having recently embarked in the dairy business.

The outcome of the recent investigation of the Bureau of Animal Industry officials at Buffalo seems to have culminated in the placing of the office of these officials at the East Buffalo stock-yards.

At a box-party at one of Philadelphia's theatres, in December, the presence was noted of City Veterinarian McNeil, of Pittsburg, Chief Meat-inspector Schrieber of Philadelphia, State Veterinarian Pearson of Harrisburg, Prof. S. J. J. Harger, of the University of Pennsylvania, Veterinary Department, and Editor Hoskins, of the JOURNAL.

Dr. John R. Mohler, of the Bureau of Animal Industry, Meat-inspection Department, stationed at Milwaukee, Wis., is on a ten days' leave of absence, visiting his home at Philadelphia.

Veterinarian Muir, of Philadelphia, fills the post of Master of his Masonic lodge during the ensuing year. In December Dr. Hoskins was selected as Junior Warden of Ivanhoe Lodge of the jurisdiction of Pennsylvania.

Dr. Harry Walter, of Wilkesbarre, was confined to his bed, from a severe attack of lumbago, in December.

